# Acute symptomatic seizures in newborns: a narrative review

**DOI:** 10.1186/s42494-024-00151-w

**Published:** 2024-02-18

**Authors:** Carlotta Spagnoli, Francesco Pisani

**Affiliations:** 1 Child Neurology Unit, Pediatric Department, Azienda USL-IRCCS Di Reggio Emilia, Reggio Emilia, 42123 Italy; 2 Child Neurology and Psychiatry Unit, Department of Human Neurosciences, Sapienza University of Rome, Rome, 00185 Italy; 3 Azienda Ospedaliero Universitaria Policlinico Umberto I, Rome, 00185 Italy

**Keywords:** Acute symptomatic, Neonatal seizures, EEG, Newborn, Outcome, Diagnosis, Etiologies

## Abstract

Acute symptomatic seizures are the main sign of neurological dysfunction in newborns. This is linked to the unique characteristics of the neonatal brain, making it hyperexcitable compared to older ages, and to the common occurrence of some forms of acquired brain injury, namely hypoxic-ischemic encephalopathy. In this narrative review we will provide an overview of neonatal seizures definition, their main underlying etiologies, diagnostic work-up and differential diagnoses, and will discuss about therapeutic options and prognostic outlook. The latest publications from the ILAE Task Force on Neonatal Seizures will be presented and discussed. Of note, they highlight the current lack of robust evidence in this field of clinical neurology. We will also report on specificities pertaining to low-and-middle income countries in terms of incidence, main etiologies and diagnosis. The possibilities offered by telemedicine and automated seizures detection will also be summarized in order to provide a framework for future directions in seizures diagnosis and management with a global perspective. Many challenges and opportunities for improving identification, monitoring and treatment of acute symptomatic seizures in newborns exist. All current caveats potentially represent different lines of research with the aim to provide better care and reach a deeper understanding of this important topic of neonatal neurology.

## Background

Seizures are the most common sign of neurological dysfunction in newborns. In a recent Italian paper, their incidence was found to correspond to 2.29/1000 live births, being higher in preterm (14.28/1000) than in full-term neonates (1.10/1000) [1]. Moreover, their incidence is inversely related to gestational age and birth weight [1, 2]. These figure are much higher, and more variable, in low/middle income countries (LMIC), where the incidence is estimated to be as high as 36–90 per 1000 live births, although with an important bias deriving from clinically-based diagnosis [3].

The proneness of the immature brain to generate seizures is linked to a series of unique developmental features increasing its excitability compared to older ages and to the higher risk of newborns to sustain brain injury. The newborn brain is characterized by an imbalance of neuronal excitation over inhibition, deriving from age-dependent expression of excitatory glutamatergic receptors, ion channels, and transporters promoting excitation, while inhibition is relatively underdeveloped [4]. In human ontogenesis, excitatory synapses develop before inhibitory ones. Glutamatergic neurons are overabundant in the newborn brain, and their receptors’ configuration allows relative hyperexcitability [5, 6]*.* In the neonatal brain GABAergic transmission has a depolarizing (excitatory) effect due to the expression of the Na–K-2Cl cotransporter 1 (NKCC1) [7–9], predating the expression of the mature potassium chloride cotransporter 2 (KCC2), responsible of the hyperpolarizing (inhibitory) effect shown at older ages under physiological conditions [9]. Additionally, in the first year of life synaptogenesis and dendritic spine density are at their peak [10, 11].

The second aspect is linked to hypoxia/ischemia being one of the most common forms of injury in the perinatal period, with developmentally regulated areas of major susceptibility and a unique ability of the immature brain to sustain and adapt to brain injury [4] (neuroplasticity).

Although this field is one of intense clinical and experimental research, many controversies and hurdles still exist in the recognition, monitoring and management of seizures in newborns.

In this narrative review, we wish to describe the definition, characteristics, diagnostic work-up, main differential diagnoses, management and outcome of acute symptomatic seizures in preterm and fullterm newborns, in light of the recent International League Against Epilepsy (ILAE) position papers, in order to provide and updated and practical framework for the clinician taking care of these fragile patients.

The search was made on PubMed by combining the following terms: “neonatal seizures”; “definition”; “classification”; “diagnosis”; “differential diagnosis”; “non-epileptic events”; “etiologies”; “therapy”; “treatment”; “outcome”. Only papers written in English and published in the last 20 years were included. This search was not intended to provide a systematic literature review, but rather to propose a general framework on the topic of acute symptomatic seizures occurring in neonates.

### Definition

The ILAE Task Force on Neonatal Seizures proposed the latest definition of a neonatal seizure as “an electrographic event with a pattern characterized by sudden, repetitive, evolving stereotyped waveforms with a beginning and end. The duration is not defined but has to be sufficient to demonstrate evolution in frequency and morphology of the discharges and needs to be long enough to allow recognition of onset, evolution, and resolution of an abnormal discharge” [12].

### Classification

**Fig. 1:**
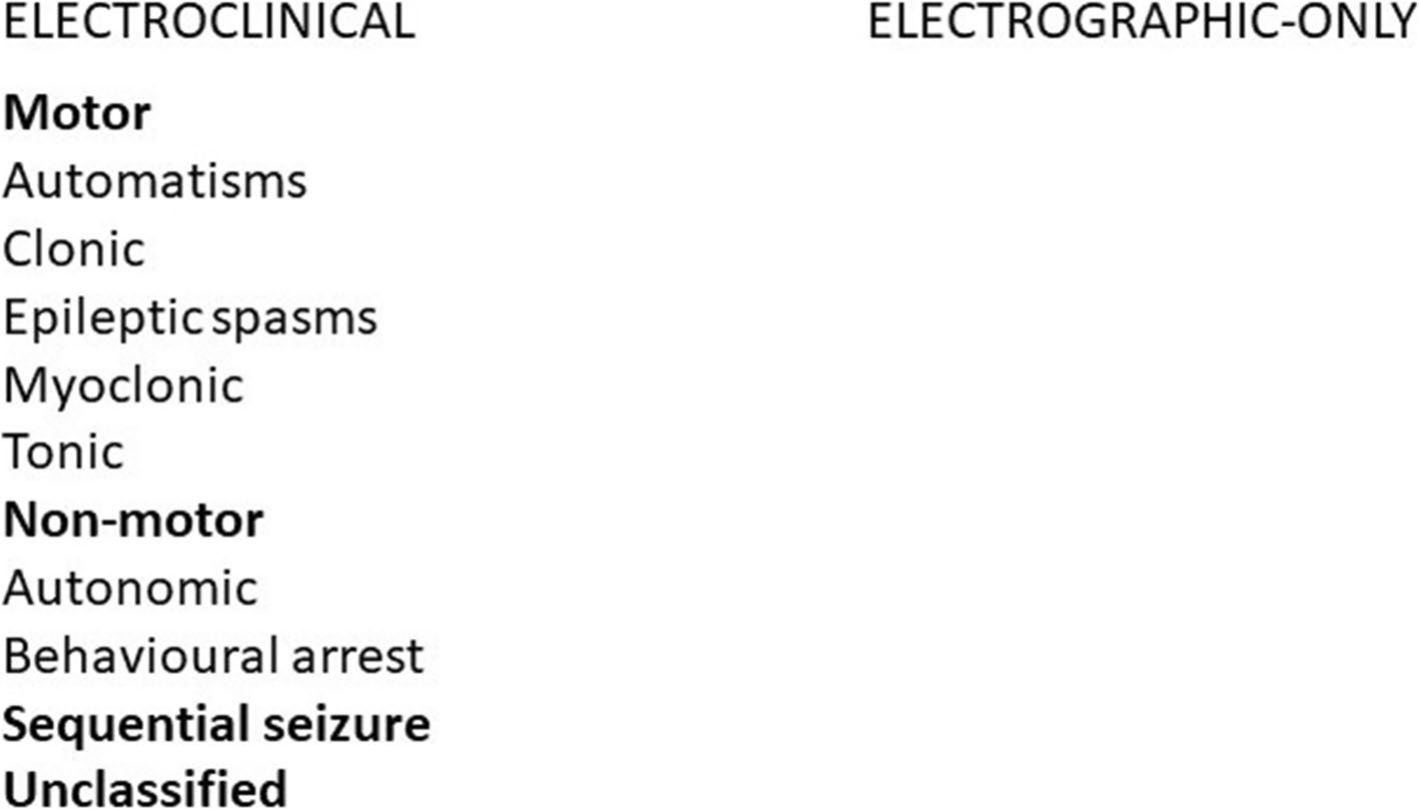
Current ILAE classification of neonatal seizures

Neonatal seizures were previously categorized as clinical-only, electroclinical, and electrographic-only [13]. However, clinical-only seizures are better categorized as non-epileptic events and therefore are no longer included in the classification. An electroclinical seizure features observable paroxysmal clinical phenomena time-locked with electroencephalographic (EEG) ictal discharges. Finally, electrographic-only seizures consist of the appearance of ictal EEG discharges without any overt clinical signs (also known as subclinical or clinically silent) [12]. In the latest classification, seizures are categorized according to their predominant clinical phenomenon into (Fig. 1): automatisms, clonic, epileptic spasms, myoclonic, tonic, autonomic (including paroxysmal cardiovascular, pupillary, gastrointestinal, sudomotor, vasomotor or thermoregulatory function abnormalities), behavioural arrest and sequential seizure. A sequential seizure is defined as “a sequence of signs, symptoms, and EEG changes at different times”, in which “no predominant feature can be determined, instead the seizure presents with a variety of clinical signs. Several features typically occur in a sequence, often with changing lateralization within or between seizures” [12].

### Diagnosis

Diagnosis of neonatal seizures, per definition, requires an EEG recording, possibly with video and polygraphy [14] to detect ictal discharges and their clinical correlates with the highest sensitivity and specificity. For an optimal documentation of each subtle clinical phenomena, the use of additional cameras has been proposed [15]. As a matter of fact, in many neonatal units worldwide, conventional EEG (or the expertise to interpret it) might not be available 24h a day, 7 days a week, and thus amplitude integrated EEG (aEEG) has become the most readily available tool for immediate bed-side interpretation, being useful as a screening tool for seizure detection, for eligibility for therapeutic hypothermia in fullterm infants with hypoxic-ischemic encephalopathy (HIE) and to assist prognostication during the rewarming phase [16]. The correct interpretation of developmental EEG features and of ictal discharges requires specialized skills and some clinical scenarios, like EEG evaluation of a severely encephalopathic newborn with depressed background, might be particularly demanding. However, in selected cases, the holistic interpretation of EEG patterns and clinical semiology might allow identification of the most plausible etiology [17], helping to prioritize investigations and treatment choices. Infants born preterm or with severe encephalopathy more often have electrographic-only seizures, especially when they are on antiseizure, sedative, or paralytic drugs. Verifying any clinical suspicion with EEG before drug administration would be the best way to investigate these babies [18].

A different scenario is present in LMIC, where the availability of EEG or aEEG is limited, making seizures diagnosis even more challenging and also reducing the expertise to interpret EEG/aEEG data. As clinical diagnosis is not reliable because of the risk of both underdiagnosis and overdiagnosis [19], the following levels of certainty in formulating the diagnosis have been proposed:

1. **Level 1: Definite seizure** (seizures confirmed on EEG, with or without clinical manifestations).
2. **Level 2: Probable seizure** (clinically assessed focal clonic or focal tonic seizures or seizures confirmed on aEEG).
3. **Level 3: Possible seizure** (clinical events suggestive of epileptic seizures other than focal clonic or focal tonic seizures).
4. **Level 4: Not seizure** (reported clinical events that do not meet case definition).
5. **Level 5: Not seizure** (clinical events evaluated by EEG and diagnosed as not a seizure) [12].

### Differential diagnosis

From an EEG point of view, the main differential diagnoses of neonatal seizures are with artefacts, which can be frequent in the busy and highly technological intensive care units, and require special care during both acquisition and interpretation [17, 20].

From a clinical point of view, paroxysmal clinical events in newborns can be due to brainstem release phenomena (i.e. generalized tonic events in preterm infants) or can correspond to a long list of paroxysmal movement disorders, which can represent “benign” self-limited (i.e. tremor, benign neonatal sleep myoclonus, startle reflex, hiccups), but also abnormal conditions (i.e. hyperekplexia, tongue fasciculations, neonatal dystonia) [21, 22], which might require dedicated diagnostic work-up and therapy, and are neither bening nor self-limited [23]. The first step to rule out an epileptic event is to perform a video-EEG, trying to capture several typical events. Paroxysmal non-epileptic events occurring in healthy newborns with normal neurological examination are expected to be self-limited and carry a favourable neurodevelopmental prognosis [24].

### Etiologies

**Fig. 2:**
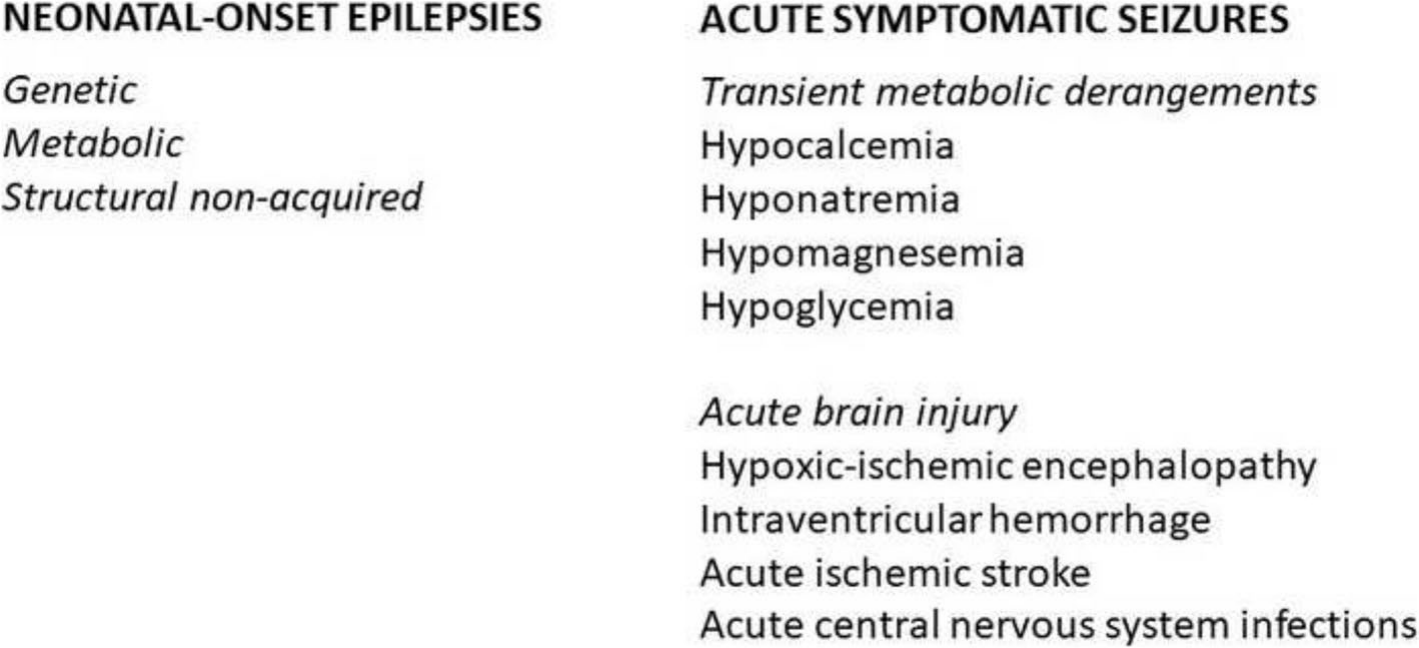
Main etiologies of seizures in newborns

Seizures in the neonate can fall within two broad categories (Fig. 2): neonatal-onset epilepsies, which can be divided into genetic, metabolic, and of structural non-acquired origin (remote symptomatic or non-provoked seizures) [25, 26] and correspond to approximately 15% of all cases [27]; acute symptomatic seizures, which are provoked seizures, and occur as a consequence of an acute event causing cortical dysfunction (approximately 85% of all cases). Among the causes for this second type of event, which is the topic of this review, we can identify transient metabolic derangements and acute brain injury as the most significant categories. In the first group, electrolytes imbalance (calcium, sodium) and hypoglycemia always need to be ruled out, because they need specific therapies and usually do not respond to conventional antiseizure medications, which are usually not necessary provided that the primary derangement is efficaciously corrected. In this category, seizures secondary to hypoglycemia (especially if prolonged and severe) have a more complex pathophysiology, as occipital lobe injury can develop and result in a risk factor for long-term epilepsy [28]. Among acute brain injuries, the most frequent etiologies include HIE (especially in the fullterm or near-term newborn [29] and intracranial hemorrhage (in particular, intraventricular haemorrhage, especially affecting the very and extremely preterm newborn [30]. Additional causes include acute ischemic stroke, which is usually a type of injury affecting the fullterm or near-term infant, and acute central nervous system infection, which can occur at any gestational age and anytime during the neonatal period, and requires prompt diagnosis and initiation of specific therapies in order to prevent further complications [31].

Epidemiology differs in high-income countries (HIC) as opposed to LMIC. While HIE is the main cause in both areas, even if the incidence is higher in LMIC, infections are the second most common cause in LMIC, whereas intracranial hemorrhage and perinatal stroke are less frequently diagnosed, possibly because of reduced access to neuroimaging. Finally, nowadays acute metabolic abnormalities are more common in LMIC because their incidence in HIC significantly decreased in the latest decades thanks to the improvements in newborns’ clinical care [3].

### Diagnostic work-up

Neonates with confirmed seizures should be thoroughly investigated, as it is not uncommon for individual patients to have more than one predisposing factor/underlying etiology [3, 31].

History taking should focus on birth, and maternal, fetal and family factors. Relevant history related to birth includes complications during, prior or immediately after delivery, such as cord prolapse/thrombosis, placental abruption, uterine rupture, non-reassuring fetal heart rate, meconium aspiration, low Apgar scores, placental abnormalities, planned home birth, operative vaginal delivery, or abnormal fetal presentation. Relevant maternal medical history can include, for esample: previous miscarriages, gestational diabetes or pre-eclampsia, infections, prenatal exposure to/withdrawal of prescription or illicit drugs, presence of inherited thrombophilias or bleeding disorders. Family history can include early sibling’s death or cases of epilepsy or other neurologic disorders [32, 33].

The timing of seizure onset can suggest specific etiologies: seizures occurring within 12–24 h after birth suggest hypoxic-ischemic encephalopathy, while seizures starting afterwards may indicate infection, hemorrhage, stroke [22] or a genetic cause.

Physical examination can be helpful in checking head size, presence of micro/macrosomia, dysmorphisms, and somatic abnormalities [32]*,* with the aim to identify clues for the underlying condition. General appearance, vital signs, level of alertness, and fontanelle characteristics might suggest bacterial meningitis, septic shock or acute intracranial hemorrhage [34]. Auscultation over the fontanelle can also disclose artero-venous malformations [33]. Skin examination can disclose findings suggestive of a congenital infection, and enables evaluation of perfusion. Acute metabolic acidosis, poor feeding, lethargy, and respiratory distress after a symptom-free period are typical presentations of inborn errors of metabolism [33, 35]. A thorough neurological examination can suggest signs of central or peripheral nervous system involvement, signs of encephalopathy, presence of focal neurological signs.

In addition, placental pathology can be used to detect signs of infection or perinatal insults [31].

Initial laboratory tests should include a complete blood count, glycemia, electrolytes [36] (to rule out: hypoglycemia, hyponatremia, hypomagnesemia, hypocalcemia), urine culture and toxicology, TORCH (toxoplasmosis, rubella cytomegalovirus, herpes simplex, and HIV) screening, metabolic screening, and ophthalmologic evaluation. CSF analysis, blood culture, C reactive protein are basically requested to rule out sepsis/meningitis/encephalitis [32].

Cranial ultrasound scan is a readily available, valuable bed-side diagnostic tool, especially for intracranial hemorrhages and their complications (i.e. post-hemorrhagic hydrocephalus), but also useful in case of arterial stroke, malformations or infections. It should be considered as a routine investigation in acute symptomatic seizures, and it should be performed as soon as possible [36]*.*

However, if available, all newborns with seizures should have brain magnetic resonance imaging (MRI) as soon as they are stable enough [18]. Brain MRI with diffusion is the gold standard, enabling identification of the main causes of seizures in newborns, such as hypoxic-ischaemic injury, arterial and venous stroke, meningitis/encephalitis, and malformations [36].

**Fig. 3:**
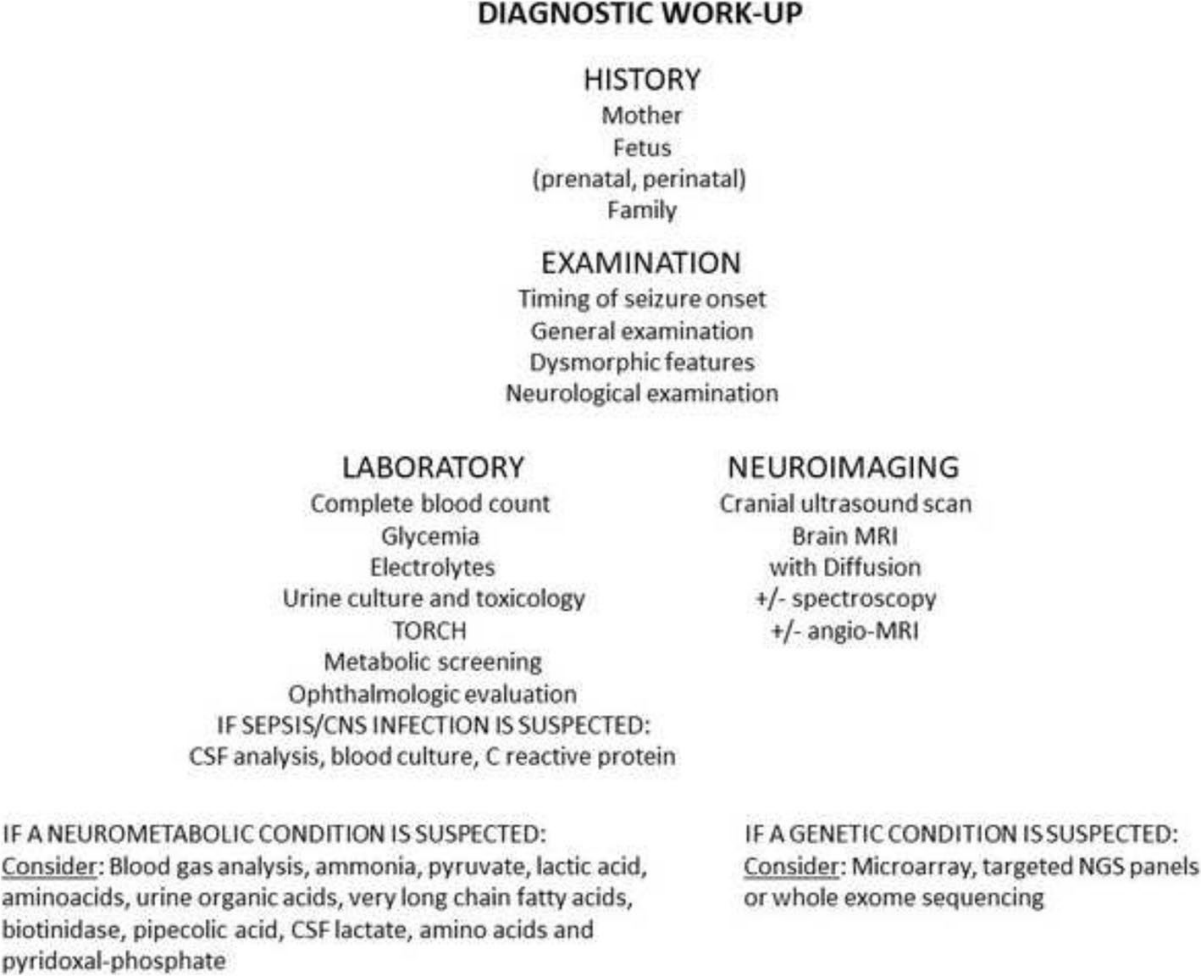
Diagnostic work-up for seizures in newborns

If the newborn has difficulty controlling seizures or exhibits additional symptoms and signs, inborn errors of metabolism and genetic neonatal-onset epilepsies should be considered, and further investigations should be tailored based on clinical suspicion/presentation. A blood gas analysis, ammonia, pyruvate, lactic acid, aminoacids and urine organic acids, very long chain fatty acids, biotinidase, pipecolic acid, CSF lactate, amino acids and pyridoxal-phosphate might all be considered on a case-by-case basis [32]. Genetic testing might include microarray, next generation sequencing with targeted panels or whole exome sequencing (WES) and whole genome sequencing (WGS) (Fig. 3) [37]. However, in HIC, due to the growing availability of NGS technologies, and their progressively reducing turnaround times and costs, directly performing WES/WGS is becoming more and more common, due to their positive cost-effectiveness profile. In fact, the clinical impact of reaching the exact diagnosis in NICUs is high, resulting in a management change in more than 50% of tested patients [38–40]. The usefulness of rapid NGS sequencing also include the opportunity to clarify confounding phenotypes in light of dual diagnoses and “solve “atypical phenotypes [41]. Directly accessing WES or WGS can allow bypassing neurometabolic testing and invasive procedures (i.e. lumbar puncture) in the early phases of the diagnostic work-up. The potential applications of NGS in newborns are also being evaluated in the field of newborn screening (NBS) programs. Although DNA sequencing cannot substitute for current conventional screening due to insufficient sensitivity and specificity, it can be beneficial during follow-up, by resolving inconclusive NBS results, distinguishing the correct disorder in cases of ambiguous NBS results, and by decreasing the numbers of follow-up samples required reducing follow-up of false positive results and by identifying disease diagnosis [42].

### Neuromonitoring

In the field of acute symptomatic seizures, the optimal timing and strategies to monitor high-risk newborns have been detailed for preterm infants and for full-term and near-term newborns with HIE [43–45]. Whenever possible, we recommend a multimodal approach [46] integrating aEEG, conventional EEG, ultrasound brain scanning, brain MRI and evoked potentials, and (ideally) near-infrared spectroscopy (NIRS) [16]. A more tailored approach is advisable for neonatal-onset epilepsies [25].

### Multichannel continuous EEG

Reliable diagnosis of seizures in newborns can only be achieved with continuous EEG monitoring of at-risk neonates [47]*.* Furthermore, screening of at-risk newborns allows optimal management, as prompt treatment initiation can result in better response to antiseizure medications compared to performing a confirmatory EEG after clinically suspected seizures [48].

High-risk neonates should be monitored for 24 h, even though the correct monitoring duration may vary according to clinical setting and previous EEG data. Once seizures are recorded, it should continue for at least 24 h after their resolution [49], in order to confirm that they truly abated with antiseizure medications [50, 51]*.*

### Amplitude-integrated EEG (aEEG)

The aEEG is a useful, non-invasive, bedside monitoring tool used to evaluate brain function (i.e. before hypothermia), background activity, and sleep-wake cycling [52] (which are all prognostic predictors) [53]. Its use in seizure diagnosis has some limitations [52]***.*** When a one-or-two-channel aEEG is used without synchronous raw EEG trace reading, sensitivity is slow (27–56%) [54]. The sensitivity and specificity of raw aEEG for the detection of neonatal seizures have been reported to range from 68% to 84% and 71% to 84%, respectively, and it is worth noting that these values strongly depend on the user’s level of experience [55]. With advanced training, up to 60–90% of newborns with seizures can be identified, although not all individual seizures might be detected, especially if the seizures are very focal, brief or distant from the scalp area covered by the electrodes [56]. Inter-observer agreement is lower than with conventional EEG, but combining a two-channel aEEG with raw EEG can enhance diagnostic accuracy [57]*.* In a prospective quality improvement cohort study, aEEG and conventional EEG were concurrently recorded, and the neonatal intensive care unit (NICU) staff interpreting aEEG had the option to contact an on-call neurologist for real-time conventional EEG interpretation [47]. This resulted in a 27% increase in correct seizure identification and avoidance of over-diagnosis in 33.3% of cases [58].

In low-resource settings, telemedicine may provide remote specialized assistance [3], as exemplified by the Protecting Brains and Saving Futures project in Brazil, where over 20 hospitals were involved in EEG or aEEG monitoring, with the on-site team being assisted by a remote specialized team, who supplied educational activities, consultation, and monitoring. Encrypted EEG data were sent to a secure cloud-based server, and access to the monitoring system, management of backup and security services were all available through authentication. However, legal and regulatory issues, but also feasibility (in terms of cost-effectiveness) would need further evaluation before such an approach can be implemented in LMIC [59]. Additionally, this is a way to initially provide and subsequently build the necessary expertise for monitoring, but equipments need to be on site, and the relative resources to be allocated.

### Automated neonatal seizure detection

Along with telemedicine, other ways to solve the issues related to the lack of around the clock availability of neonatal EEG expertise in NICU’s, are represented by automated detection systems and artificial intelligence [60].

Automated detection systems can assist in background grading and/or seizures recognition, either with video, EEG or other biological signals’ analysis. The topic has been recently reviewed [61]. Among EEG-based systems, different approaches can be identified: heuristic algorithms (based on an empirical definition of rules, thresholds, and parameters, and on the search for variations in signal trends [62–64]), data-driven algorithsms (based on machine learning techniques, in which features, rules and thresholds are learnt during data acquisition in the training phase) [65–67], deep-learning algorithms (which extract information from data and learn abstract representations features [68–70]), electrocardiogram (ECG)-based systems (assessing changes in inter-beat time intervals, or heart rate variability – HRV) [71–75], and video-based systems [76–83], which are not intended to substitute interpretation of EEG signal but might be integrated into multiparametric detection systems.

So far a single multicentre, randomized, controlled trial has been published on the subject. It compared conventional EEG plus the Algorithm for Neonatal Seizure Recognition (ANSeR) versus conventional EEG monitoring alone. ANSeR was linked to the EEG monitor, and displayed a seizure probability trend in real time. While the algorithm did not improve identification of individual newborns with seizures, it improved recognition of seizure hours (i.e. the total duration of seizures was better clarified). The authors thus hypothesized better advantages in less experienced centres [67]. Some of these algorithms are commercially available [82–85]. Artificial intelligence is being increasingly proposed as an instrument to overcome the complexity of interpreting EEG for neonatal seizures diagnosis. In a recent study, EEG is converted to sound, so that the perceptual characteristics of seizures can be heard and interpreted by medical personnel with little or no expertise in neonatal neurophysiology, with an accuracy similar to that of trained neurophysiologists and higher than that of artificial intelligence alone [86].

### Therapy

A part from correcting acute electrolytic and metabolic derangements, clinical practice on the treatment of neonatal seizures with antiseizure medications may differ according to subspecialty (i.e. neonatologists versus pediatric or neonatal neurologists), and according to seizure etiology, reflecting the lack of robust evidence from the literature and the persistence of knowledge gaps, especially for the management of preterm infants*.* A multicentre Italian survey among pediatric neurologists working in third level NICUs documented that the most commonly suggested sequence of antiseizure medications in case of acute symptomatic seizures was: phenobarbital (79%), phenytoin (58%), midazolam (42%), and levetiracetam (42%), while for neonatal seizures due to a genetic or structural epilepsy, the concordance among pediatric neurologists was lower [87]*.*

This can be partially explained by the many controversies surrounding phenobarbital, which is only effective in approximately half of the cases [88], causes electroclinical uncoupling and can have relevant adverse effects, such as hypotension, sedation and respiratory suppression [89] which might be particularly relevant in critically-ill newborns [90]. Finally, experimental data suggest a negative effect on neuronal apoptosis [91] and synaptic maturation [92].

These considerations have led to increasing off-label prescription, especially of levetiracetam [93], thanks to its good safety profile [94], although efficacy seems to be lower than that of phenobarbital [90]*.* In particular, in one study recruiting 38 newborns, receiving levetiracetam as first-line, 19 newborns had one dose of phenobarbital and 3 received two phenobarbital doses due to lack of seizure control on levetiracetam. At the end of the first week, however, 30 neonates were seizure free on levetiracetam and 27 were still seizure free at four weeks [95]. A multicenter, randomized, blinded, controlled trial randomly assigning newborns to phenobarbital or levetiracetam as first-line treatment documented 80% seizure freedom for 24 h on phenobarbital versus 28% on levetiracetam, with a 7.5% improvement in efficacy with levetiracetam dose escalation to 60 mg/kg [89]. Subsequently, other groups documented more encouraging results [96]. A systematic review and meta-analysis of studies evaluating levetiracetam as the first-line treatment of neonatal seizures in preterm and full-term infants analyzed 14 studies assessing 1188 newborns. Pooled efficacy with levetiraetam from observational studies was 45%, while meta-analysis of randomized controlled trials comparing it with phenobarbital showed equal effectiveness, but lower risk of short-term adverse events with levetiracetam [97]. As acute symptomatic seizures tend to spontaneously abate with time, it is noteworthy to also investigate response rate as add-on. One recent retrospective study evaluated efficacy of levetiracetam administered as a 2nd-to-4th-line to near term newborns not responding to first-line phenobarbital. Efficacy was defined as > 80% seizure reduction. This was obtained in 17% of newborns on levetiracetam, versus 23% after midazolam and 92% after lidocaine [98], arguing in favour of further, prospective research involving homogeneous populations in terms of gestational ages and etiologies, and with definite protocols for seizures monitoring.

**Fig. 4:**
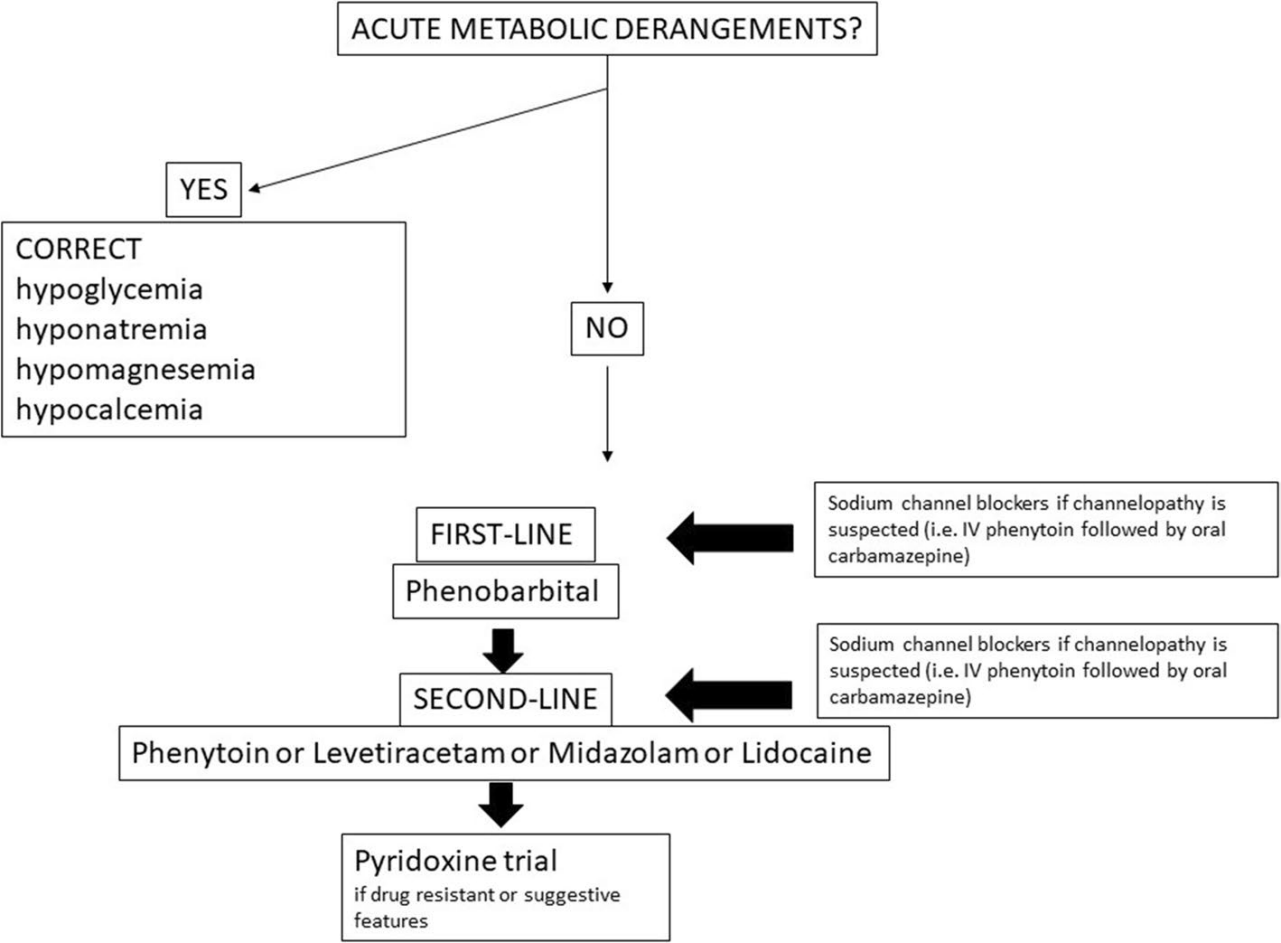
Flow-chart for seizures treatment (modified from Gotman J, et al [84])

Recently, the ILAE Task Force on Neonatal Seizures published evidence-based and consensus-based recommendations for treatment, which can be summarized as follows. Phenobarbital should be the first-line treatment, irrespective of seizures etiology, unless a channelopathy is suspected, in which case sodium channel blockers such as phenytoin and carbamazepine should be administered. If neonates do not respond to the first-line drug, phenytoin, levetiracetam, midazolam, or lidocaine may be used as a second-line [88, 89, 99]*.* Levetiracetam was recommended (based on expert opinion) in newborns with cardiac diseases, due to potential for cardiotoxic effects of alternative second-line drugs (i.e. phenytoin, lidocaine) [100]. Based on expert opininon, pyridoxine might be trialed if clinical features of vitamin B6-dependent epilepsy are present and when seizures do not respond to second-line medications. These recommendations highlight the current lack of robust evidence on the therapeutic management of seizures in newborns, especially beyond firt-line management (Fig. 4).

According to a recent Cochrane review, if seizures cease after the first loading dose of phenobarbital, maintenance therapy compared to no maintenance may have little or no effect on mortality before discharge, mortality or neurodevelopmental disability by 18–24 months and epilepsy post-discharge [101]*.* In neonates with HIE, treatment of both clinical and electrographic-only seizures compared to clinical seizures alone may have little or no effect on seizure burden during hospitalisation, mortality before discharge and epilepsy post-discharge [101]*.* However, caution should be expresses, as the level of evidence is low and some literature data indeed seem to support the notion that electrographic-only seizures can be just as detrimental as electroclinical seizures [102].

Additionally, prolonged or recurrent seizures might worsen brain injury beyond that of the underlying etiology [103, 104], a notion that is sustained by both experimental and clinical data [103–107]. A recent study documented a significant negative correlation between seizure burden and developmental scores, and this association was stronger for HIE and stroke [108]. Prompt recognition of seizures can result in higher drug response [88, 89]*.* As an example, neonates treated within 1 h of seizure onset had lower seizure burden and fewer seizures in the following 24 h, suggesting that the impact of antiseizure medications on seizure burden is time-critical [66]. However, the optimal timepoint for treatment initiation is yet to be defined [90]. A cumulative electrographic seizure burden of more than 30 s/h was proposed as an entry criterion in therapeutic trials [109].

Factors associated with lack of response to antiseizure medications have been seldom investigated. They include higher seizure frequency, particularly status epilepticus [110], electrographic-only seizures, and abnormal EEG background [111]*,* higher mean seizure score and higher degrees of brain MRI injury (white matter, cortex, and watershed regions) [112]*.*

### Outcome

Acute symptomatic seizures in newborns are frequently followed by the occurrence of various unfavorable outcomes, including death, cerebral palsy, epilepsy, intellectual disability, vision and hearing impairment, and microcephaly [103, 112–115].

Mortality is higher in preterm (32–35%) than in full-term newborns (5.4–15%) [103, 116–118]. In a systematic review on papers investigating the outcome of preterm infants with seizures published in the 2000’s, we found a 11.3–38.9% occurrence of epilepsy, a 12–84.6% of cerebral palsy, and a 20–42.7% of intellectual disability or developmental delay. When a comparison group without seizures was present, outcome was worse in infants with seizures [119].

Outcome clearly changes according to birth weight and gestational age. Mortality of preterm infants with seizures increases in patients with a birth weight < 1000 g and a gestational age < 28 weeks [120]*.* Among preterm infants with seizures evaluated at the mean age of 6.2 ± 2.0, years, 44.4% of the very low birth weight (VLBW) group and 71.4% of the low birth weight (LBW) group showed intellectual impairment, while cerebral palsy was present in 22% of VLBW and 42.9% of LBW infants. Postneonatal epilepsy was present in 11.1% of VLBW infants and 28.5% of LBW infants [121].

Data on full-term newborns report 17% of cerebral palsy [122], 35% of intellectual disability [123], and 10–15% of epilepsy [124] in NICU-based series, which collect information on the most severely affected newborns.

Epilepsy most often starts early, occurring within the first year of life in 68.5% of cases [125], except in case of perinatal arterial ischemic stroke, where the age at the first post-neonatal seizure ranges from 1 to 10 years, but the latent period can be as long as 15 years [126]. The rate of epilepsy following perinatal stroke is around 16%, and infarct location (right middle cerebral artery; multiple involved territories) is a negative independent predictor [127]*.*

In full-term and near-term infants with moderate-to-severe HIE, the use of therapeutic hypothermia has significantly changed the outcome of these newborns. In fact it is associated with an increase in survival with normal neurological exam, and it significantly lowers the risk of cerebral palsy, moderate/severe disability and epilepsy [128]. However, still almost half either die or suffer from severe neurodevelopmental disabilities, while 40% have a normal neurodevelopmental outcome [129]. Neurologic sequelae can range from mild to disabling. Cerebral palsy develops in 13% of cases [130], while an IQ score below 70 is found in 96% of children with cerebral palsy and in 9% without [131]. The percentage of infants with abnormal outcome is higher in those presenting with neonatal seizures: 62% versus 39% [105].

The current prognostic outlook of seizures in LMIC has different characteristics and specific challenges compared to high income countries. These include (among others) mortality rates, compliance to scheduled pregnancy evaluations, epidemiology regarding etiologies, availability of EEG and frequent clinical definition of seizures. Mortality rate differs according to studied populations and geographical areas. Mortality in the neonatal period is reported to vary [132–136] between 7.8% [2] and 32.51% [137]*.* The occurrence of additional deaths after the neonatal period was reported to correspond to 3.94% in [137] and to 9.1% in [133]. Interestingly, a study from a rural area in Kenya found no differences based on the occurrence of seizures, but a different profile risk based on birth weight, with a detrimental role of seizures in newborns over 2500 g [133], possibly outlining the predominance of other (stronger) determinants of unfavourble outcome in the low birth weight group, pointing out to a need for addressing different management objectives in these babies. At discharge, 10–13% of the surviving neonates have an abnormal neurological exam [132, 133], and 8.4% severe neurologic deficits [135]. In a study performed in India, 26.1% of survivors had abnormal neurological examination at discharge, either in the form of abnormal tone (20.3%), reflexes (14.4%) or consciousness (7.2%) [134].

Identifying the main factors determining outcome is crucial for the correct management of newborns and correct counseling to the families. Among the most important prognostic factors we can include: antenatal—including placental—factors (i.e. chorioamnionitis) and multiple perinatal factors, such as gestational age, Apgar scores, prolonged resuscitation, birth weight, etiology (HIE and intraventricular hemorrage [IVH] carrying the worst prognosis), severity and patterns of brain injury, neurological examination, presence of a congenital heart disease [138] and abnormal background EEG [139–142].

However, among these, the most important roles are played by etiology, and location and severity of brain injury. In hypoxic-ischemic encephalopathy, brain MRI patterns of injury predict the severity and type of neurodevelopmental sequelae [143–145]: basal ganglia–thalamic and brainstem injury are often associated with the severest impairments (cerebral palsy, cognitive impairments and epilepsy), whereas watershed type of injury mostly affects cognitive development [143–147].

However, seizure-related factors are also critical. Longer seizure duration, higher seizure burden [143] and especially the occurrence of status epilepticus are important determinants [105, 140, 142, 148]. In detail, spreading of the ictal discharges to the contralateral hemisphere, status epilepticus [118, 141], a family history of epilepsy, abnormal neurological examination at discharge [149], poor response to anticonvulsants [124], and the use of at least two antiseizure medications represent risk factors for later epilepsy [149]. The latter variable also relates to severe neurodevelopmental delay in fullterm infants [150] and to the risk of death and poor outcome in hypoxic-ischemic encephalopathy [151, 152]*.* Additionally, in infants with hypoxic-ischemic encephaloapthy, although the presence of seizures per se was not associated with abnormal outcome, a total seizure burden exceeding 40 min and a maximum hourly seizure burden of more than 13 min were associated with unfavourable outcome, independently of electrographic grade of HIE or therapeutic hypothermia*.* In any case, it would be useful to collect further data on outcome determinants in newborns with specific etiologies and possibly controlled for degree of brain injury, and to study populations of newborns outside the better known subgroup of newborns with hypoxisc-ischemic encephalopathy.

## Conclusions

Seizures in newborns are a frequent phenomenon, and need to be: correctly interpreted, documented and monitored; promptly and efficaciously treated; thoroughly investigated. Each of these steps is time-consuming, and requires specialized skills and dedicated resources (in terms of personnel and devices). Thus, delivering the best possible care to high-risk and critically ill newborns is challenging. However, this is critical to improve survival and long-term outcome. These challenges can be dealt with at different levels: from promoting education and building specialized expertise, to developing telemedicine programs; from organizing clinical trials in order to collect robust evidence, to supporting preclinical research on many controversial topics. In the mean time, the international effort to uniform glossaries, taxonomy and monitoring and to provide state-of-the-art guidance for treatment is an important way to disseminate better standards of care.

## Data Availability

Not applicable.

## Abbreviations

aEEG: Amplitude-integrated EEG
ANSeR: Algorithm for Neonatal Seizure Recognition
CSF: Cerebrospinal fluid
ECG: Electrocardiogram
EEG: Electroencephalogram
HRV: Heart Rate Variability
ILAE: International League Against Epilepsy
IVH: Intraventricular hemorrhage
LMIC: Low/middle income countries
KCC2: Potassium Chloride Cotransporter 2
NKCC1: Na–K-2Cl cotransporter 1
HIC: High-income countries
HIE: Hypoxic-ischemic encephalopathy
MRI: Magnetic resonance imaging
NICU: Neonatal intensive care unit
NIRS: Near-infrared spectroscopy

## References

[1] Pisani F, Facini C, Bianchi E, Giussani G, Piccolo B, Beghi E. Incidence of neonatal seizures, perinatal risk factors for epilepsy and mortality after neonatal seizures in the province of Parma Italy. Epilepsia. 2018;59(9):1764–73. 30132843 10.1111/epi.14537

[2] Yan K, Cheng G, Zhou W, Xiao F, Zhang C, Wang L, et al. Incidence of neonatal seizures in china based on electroencephalogram monitoring in neonatal neurocritical care units. JAMA Netw Open. 2023;6(7):e2326301. 37505497 10.1001/jamanetworkopen.2023.26301 PMC10383014

[3] Vegda H, Krishnan V, Variane G, Bagayi V, Ivain P, Pressler RM. Neonatal seizures-perspective in low-and middle-income countries. Indian J Pediatr. 2022;89(3):245–53. 35050459 10.1007/s12098-021-04039-2 PMC8857130

[4] Jensen FE. Developmental factors regulating susceptibility to perinatal brain injury and seizures. Curr Opin Pediatr. 2006;18(6):628–33. 17099361 10.1097/MOP.0b013e328010c536

[5] Rakhade SN, Jensen FE. Epileptogenesis in the immature brain: emerging mechanisms. Nat Rev Neurol. 2009;5(7):380–91. 19578345 10.1038/nrneurol.2009.80 PMC2822660

[6] Sanchez RM, Jensen FE. Maturational aspects of epilepsy mechanisms and consequences for the immature brain. Epilepsia. 2001;42(5):577–85. 11380563 10.1046/j.1528-1157.2001.12000.x

[7] Dzhala VI, Staley KJ. Excitatory actions of endogenously released GABA contribute to initiation of ictal epileptiform activity in the developing hippocampus. J Neurosci. 2003;23(5):1840–6. 12629188 10.1523/JNEUROSCI.23-05-01840.2003 PMC6741948

[8] Dzhala VI, Talos DM, Sdrulla DA, Brumback AC, Mathews GC, Benke TA, et al. NKCC1 transporter facilitates seizures in the developing brain. Nat Med. 2005;11(11):1205-13.20. 16227993 10.1038/nm1301

[9] Khazipov R, Khalilov I, Tyzio R, Morozova E, Ben-Ari Y, Holmes GL. Developmental changes in GABAergic actions and seizure susceptibility in the rat hippocampus. Eur J Neurosci. 2004;19(3):590–600. 14984409 10.1111/j.0953-816x.2003.03152.x

[10] Huttenlocher PR, de Courten C, Garey LJ, Van der Loos H. Synaptogenesis in human visual cortex–evidence for synapse elimination during normal development. Neurosci Lett. 1982;33(3):247–52. 7162689 10.1016/0304-3940(82)90379-2

[11] Takashima S, Chan F, Becker LE, Armstrong DL. Morphology of the developing visual cortex of the human infant: a quantitative and qualitative Golgi study. J Neuropathol Exp Neurol. 1980;39(4):487–501. 7217997 10.1097/00005072-198007000-00007

[12] Pressler RM, Cilio MR, Mizrahi EM, Moshé SL, Nunes ML, Plouin P, et al. The ILAE classification of seizures and the epilepsies: Modification for seizures in the neonate. Position paper by the ILAE Task Force on Neonatal Seizures. Epilepsia. 2021;62(3):615–28. 33522601 10.1111/epi.16815

[13] Mizrahi EM, Kellaway P. Characterization and classification of neonatal seizures. Neurology. 1987;37(12):1837–44. 3683874 10.1212/wnl.37.12.1837

[14] Tsuchida TN, Wusthoff CJ, Shellhaas RA, Abend NS, Hahn CD, Sullivan JE, et al. American clinical neurophysiology society standardized EEG terminology and categorization for the description of continuous EEG monitoring in neonates: report of the American Clinical Neurophysiology Society critical care monitoring committee. J Clin Neurophysiol. 2013;30(2):161–73. 23545767 10.1097/WNP.0b013e3182872b24

[15] Pisani F, Pavlidis E, Cattani L, Ferrari G, Raheli R, Spagnoli C. Optimizing detection rate and characterization of subtle paroxysmal neonatal abnormal facial movements with multi-camera video electroencephalogram recordings. Neuropediatrics. 2016;47(3):169–74. 27111027 10.1055/s-0036-1582245

[16] El-Dib M, Abend NS, Austin T, Boylan G, Chock V, Cilio MR, et al. Neuromonitoring in neonatal critical care part I: neonatal encephalopathy and neonates with possible seizures. Pediatr Res. 2023;94(1):64–73. 36476747 10.1038/s41390-022-02393-1

[17] Pisani F, Spagnoli C. EEG in neonatal seizures: where to look and what to see. Expert Rev Neurother. 2022;22(11–12):963–79. 36637240 10.1080/14737175.2022.2169132

[18] Ramantani G, Schmitt B, Plecko B, Pressler RM, Wohlrab G, Klebermass-Schrehof K, et al. Neonatal seizures-are we there yet? Neuropediatrics. 2019;50(5):280–93. 31340400 10.1055/s-0039-1693149

[19] Murray DM, Boylan GB, Ali I, Ryan CA, Murphy BP, Connolly S. Defining the gap between electrographic seizure burden, clinical expression and staff recognition of neonatal seizures. Arch Dis Child Fetal Neonatal Ed. 2008;93(3):F187–91. 17626147 10.1136/adc.2005.086314

[20] Malfilâtre G, Mony L, Hasaerts D, Vignolo-Diard P, Lamblin MD, Bourel-Ponchel E. Technical recommendations and interpretation guidelines for electroencephalography for premature and full-term newborns. Neurophysiol Clin. 2021;51(1):35–60. 33168466 10.1016/j.neucli.2020.10.005

[21] Facini C, Spagnoli C, Pisani F. Epileptic and non-epileptic paroxysmal motor phenomena in newborns. J Matern Fetal Neonatal Med. 2016;29(22):3652–9. 26918366 10.3109/14767058.2016.1140735

[22] Orivoli S, Facini C, Pisani F. Paroxysmal nonepileptic motor phenomena in newborn. Brain Dev. 2015;37(9):833–9. 25687201 10.1016/j.braindev.2015.01.002

[23] Huntsman RJ, Lowry NJ, Sankaran K. Nonepileptic motor phenomena in the neonate. Paediatr Child Health. 2008;13(8):680–4. 19436521 10.1093/pch/13.8.680 PMC2606074

[24] Romano C, Giacchi V, Mauceri L, Pavone P, Taibi R, Gulisano M, et al. Neurodevelopmental outcomes of neonatal non-epileptic paroxysmal events: a prospective study. Dev Med Child Neurol. 2021;63(3):343–8. 33336794 10.1111/dmcn.14784

[25] Pisani F, Spagnoli C, Fusco C. EEG Monitoring of the epileptic newborn. Curr Neurol Neurosci Rep. 2020;20(4):6. 32166392 10.1007/s11910-020-1027-7

[26] Pisani F, Spagnoli C, Falsaperla R, Nagarajan L, Ramantani G. Seizures in the neonate: a review of etiologies and outcomes. Seizure. 2021;85:48–56. 33418166 10.1016/j.seizure.2020.12.023

[27] Shellhaas RA, Wusthoff CJ, Tsuchida TN, Glass HC, Chu CJ, Massey SL, et al. Profile of neonatal epilepsies: characteristics of a prospective US cohort. Neurology. 2017;89(9):893–9. 28733343 10.1212/WNL.0000000000004284 PMC5577964

[28] Wong DST, Poskitt KJ, Chau V, Miller SP, Roland E, Hill A, et al. Brain injury patterns in hypoglycemia in neonatal encephalopathy. AJNR Am J Neuroradiol. 2013;34:1456–61. 23436054 10.3174/ajnr.A3423 PMC8051482

[29] Soul JS. Acute symptomatic seizures in term neonates: Etiologies and treatments. Semin Fetal Neonatal Med. 2018;23(3):183–90. 29433814 10.1016/j.siny.2018.02.002 PMC6026476

[30] Pisani F, Spagnoli C. Acute symptomatic neonatal seizures in preterm neonates: etiologies and treatments. Semin Fetal Neonatal Med. 2018;23(3):191–6. 29467102 10.1016/j.siny.2017.12.003

[31] Ziobro J, Shellhaas RA. Neonatal seizures: diagnosis, etiologies, and management. Semin Neurol. 2020;40(2):246–56. 32143234 10.1055/s-0040-1702943

[32] Kim EH, Shin J, Lee BK. Neonatal seizures: diagnostic updates based on new definition and classification. Clin Exp Pediatr. 2022;65(8):387–97. 35381171 10.3345/cep.2021.01361 PMC9348949

[33] Krawiec C, Muzio MR. Neonatal seizure. In: StatPearls. Treasure Island: StatPearls Publishing; 2023. 32119422

[34] Hallberg B, Blennow M. Investigations for neonatal seizures. Semin Fetal Neonatal Med. 2013;18(4):196–201. 23680099 10.1016/j.siny.2013.03.001

[35] Shellhaas R. Clinical features, evaluation, and diagnosis of neonatal seizures. UpToDate https://www.uptodate.com/contents/clinical-features-evaluation-and-diagnosis-of-neonatal-seizures. Accessed 20 Nov 2023.

[36] Glass HC. Neonatal seizures: advances in mechanisms and management. Clin Perinatol. 2014;41:177–90. 24524454 10.1016/j.clp.2013.10.004 PMC3925308

[37] Akbari H, Sunderraj A, Sanchez-Pinto N, Berg AT, George AL Jr, Pardo AC. Genetic testing and hospital length of stay in neonates with epilepsy. Pediatr Neurol. 2022;133:30–3. 35751960 10.1016/j.pediatrneurol.2022.05.011 PMC9484310

[38] Clark MM, Hildreth A, Batalov S, Ding Y, Chowdhury S, Watkins K, et al. Diagnosis of genetic diseases in seriously ill children by rapid whole-genome sequencing and automated phenotyping and interpretation. Sci Transl Med. 2019;11(489):eaat6177. 31019026 10.1126/scitranslmed.aat6177 PMC9512059

[39] Elliott AM, du Souich C, Lehman A, Guella I, Evans DM, Candido T, et al. RAPIDOMICS: rapid genome-wide sequencing in a neonatal intensive care unit-successes and challenges. Eur J Pediatr. 2019;178(8):1207–18. 31172278 10.1007/s00431-019-03399-4

[40] Freed AS, Clowes Candadai SV, Sikes MC, Thies J, Byers HM, Dines JN, et al. The impact of rapid exome sequencing on medical management of critically ill children. J Pediatr. 2020;226:202-12.e1. 32553838 10.1016/j.jpeds.2020.06.020 PMC7736066

[41] Muriello M. Exome and whole genome sequencing in the neonatal intensive care unit. Clin Perinatol. 2022;49(1):167–79. 35209999 10.1016/j.clp.2021.11.018

[42] Woerner AC, Gallagher RC, Vockley J, Adhikari AN. The use of whole genome and exome sequencing for newborn screening: challenges and opportunities for population health. Front Pediatr. 2021;9:663752. 34350142 10.3389/fped.2021.663752 PMC8326411

[43] Lamblin MD, Walls Esquivel E, André M. The electroencephalogram of the full-term newborn: review of normal features and hypoxic-ischemic encephalopathy patterns. Neurophysiol Clin. 2013;43(5–6):267–87. 24314754 10.1016/j.neucli.2013.07.001

[44] Nguyen The Tich S, d’Allest AM, Villepin AT, de Belliscize J, Walls-Esquivel E, Salefranque F, et al. Pathological features of neonatal EEG in preterm babies born before 30 weeks of gestationnal age. Neurophysiol Clin. 2007;37:325–70. 18063234 10.1016/j.neucli.2007.10.001

[45] Chang T, Tsuchida TN. Conventional (continuous) EEG monitoring in the NICU. Curr Pediatr Rev. 2014;10:2–10. 25055858 10.2174/157339631001140408115626

[46] Pisani F, Spagnoli C. Monitoring of newborns at high risk for brain injury. Ital J Pediatr. 2016;42(1):48. 27180227 10.1186/s13052-016-0261-8 PMC4867092

[47] Boylan GB, Stevenson NJ, Vanhatalo S. Monitoring neonatal seizures. Semin Fetal Neonatal Med. 2013;18(4):202–8. 23707519 10.1016/j.siny.2013.04.004

[48] Wusthoff CJ, Sundaram V, Abend NS, Massey SL, Lemmon ME, Thomas C, et al. Seizure control in neonates undergoing screening vs confirmatory EEG monitoring. Neurology. 2021;97:e587–96. 34078719 10.1212/WNL.0000000000012293 PMC8424499

[49] Shellhaas RA, Chang T, Tsuchida T, Scher MS, Riviello JJ, Abend NS, et al. The American Clinical Neurophysiology Society's Guideline on Continuous Electroencephalography Monitoring in Neonates. J Clin Neurophysiol. 2011;28(6):611–7. 10.1097/WNP.0b013e31823e96d7 22146359

[50] Shellhaas RA. Continuous long-term electroencephalography: the gold standard for neonatal seizure diagnosis. Semin Fetal Neonatal Med. 2015;20:149–53. 25660396 10.1016/j.siny.2015.01.005

[51] Okumura A. Electroencephalography in neonatal epilepsies. Pediatr Int. 2020;62:1019–28. 32153072 10.1111/ped.14227

[52] Variane GFT, Rodrigues DP, Pietrobom RFR, França CN, Netto A, Magalhães M. Newborns at high risk for brain injury: the role of the amplitude-integrated electroencephalography. J Pediatr (Rio J). 2022;98(6):565–71. 34986412 10.1016/j.jped.2021.10.008 PMC9617284

[53] de Vries LS, Hellström-Westas L. Role of cerebral function monitoring in the newborn. Arch Dis Child Fetal Neonatal Ed. 2005;90(3):F201–7. 15846008 10.1136/adc.2004.062745 PMC1721888

[54] Shah DK, Mackay MT, Lavery S, Watson S, Harvey AS, Zempel J, et al. Accuracy of bedside electroencephalographic monitoring in comparison with simultaneous continuous conventional elec-troencephalography for seizure detection in term infants. Pediatrics. 2008;121(6):1146–54. 10.1542/peds.2007-1839 18519484

[55] Frenkel N, Friger M, Meledin I, Berger I, Marks K, Bassan H, et al. Neonatal seizure recognition–comparative study of continuous-amplitude integrated EEG versus short conventional EEG recordings. Clin Neurophysiol. 2011;122(6):1091–1097.21216190. 21216190 10.1016/j.clinph.2010.09.028

[56] DeLaGarza-Pineda O, Mailo JA, Boylan G, Chau V, Glass HC, Mathur AM, et al. Management of seizures in neonates with neonatal encephalopathy treated with hypothermia. Semin Fetal Neonatal Med. 2021;26(4): 101279. 34563467 10.1016/j.siny.2021.101279

[57] Rakshasbhuvankar A, Paul S, Nagarajan L, Ghosh S, Rao S. Amplitude integrated EEG for detection of neonatal seizures: a systematic review. Seizure. 2015;33:90–8. 26456517 10.1016/j.seizure.2015.09.014

[58] Mendelsohn R, Lemyre B, Webster RJ, Mabilangan K, Bulusu S, Pohl D. Real-time detection of neonatal seizures improves with on demand EEG Interpretation. Clin Neurophysiol. 2022;143:166–71. 36115811 10.1016/j.clinph.2022.08.017

[59] Krishnan V, Kumar V, Variane GFT, Carlo WA, Bhutta ZA, Sizonenko S, et al. Need for more evidence in the prevention and management of perinatal asphyxia and neonatal encephalopathy in low and middle-income countries: a call for action. Semin Fetal Neonatal Med. 2021;26(5):101271. 34330679 10.1016/j.siny.2021.101271 PMC8650826

[60] Olmi B, Frassineti L, Lanata A, Manfredi C. Automatic detection of epileptic seizures in neonatal intensive care units through EEG, ECG and video recordings: a survey. IEEE Access. 2021;9:138174–91.

[61] Liu A, Hahn JS, Heldt GP, Coen RW. Detection of neonatal seizures through computerized EEG analysis. Electroencephalogr Clin Neurophysiol. 1992;82(1):30–7. 1370141 10.1016/0013-4694(92)90179-l

[62] Gotman J, Flanagan D, Zhang J, Rosenblatt B. Automatic seizure detection in the newborn: methods and initial evaluation. Electroencephalogr Clin Neurophysiol. 1997;103(3):356–62. 9305282 10.1016/s0013-4694(97)00003-9

[63] Deburchgraeve W, Cherian PJ, De Vos M, Swarte RM, Blok JH, Visser GH, et al. Automated neonatal seizure detection mimicking a human observer reading EEG. Clin Neurophysiol. 2008;119(11):2447–54. 18824405 10.1016/j.clinph.2008.07.281

[64] Thomas EM, Temko A, Lightbody G, Marnane WP, Boylan GB. Gaussian mixture models for classification of neonatal seizures using EEG. Physiol Meas. 2010;31(7):1047–64. 20585148 10.1088/0967-3334/31/7/013 PMC3428723

[65] Temko A, Thomas E, Marnane W, Lightbody G, Boylan G. EEG-based neonatal seizure detection with support vector machines. Clin Neurophysiol. 2011;122(3):464–73. 20713314 10.1016/j.clinph.2010.06.034 PMC3036797

[66] Pavel AM, Rennie JM, de Vries LS, Blennow M, Foran A, Shah DK, et al. A machine-learning algorithm for neonatal seizure recognition: a multicentre, randomised, controlled trial. Lancet Child Adolesc Health. 2020;4(10):740–9. 32861271 10.1016/S2352-4642(20)30239-X PMC7492960

[67] Ansari AH, Cherian PJ, Caicedo A, Naulaers G, De Vos M, Van Huffel S. Neonatal Seizure Detection Using Deep Convolutional Neural Networks. Int J Neural Syst*.* 2019;29(4):1850011. 10.1142/S0129065718500119 29747532

[68] O’Shea A, Lightbody G, Gordon G, Boylan G, Temko A. Neonatal seizure detection from raw multi-channel EEG using a fully convolutional architecture. Neural Netw. 2020;123:12–25. 31821947 10.1016/j.neunet.2019.11.023

[69] Tanveer MA, Khan MJ, Sajid H, Naseer N. Convolutional neural networks ensemble model for neonatal seizure detection. J Neurosci Methods. 2021;358:109197. 33864835 10.1016/j.jneumeth.2021.109197

[70] Malarvili MB, Mesbah M, Boashash B. Time-Frequency Analysis of Heart Rate Variability for Neonatal Seizure Detection. EURASIP J Adv Signal Process. 2007;2007(1):67. 16623224

[71] Greene BR, de Chazal P, Boylan GB, Connolly S, Reilly R. Electrocardiogram based neonatal seizure detection. IEEE Trans Biomed Eng. 2006;54(4):673–82. 10.1109/TBME.2006.890137 17405374

[72] Doyle OM, Temko A, Marnane W, Lightbody G. Heart rate based automatic seizure detection in the newborn. Med Eng Phys. 2010;32(8):829–39. 20594899 10.1016/j.medengphy.2010.05.010

[73] Statello R, Carnevali L, Sgoifo A, Miragoli M, Pisani F. Heart rate variability in neonatal seizures: Investigation and implications for management. Neurophysiol Clin. 2021;51(6):483–92. 34774410 10.1016/j.neucli.2021.10.002

[74] Statello R, Carnevali L, Alinovi D, Pisani F, Sgoifo A. Heart rate variability in neonatal patients with seizures. Clin Neurophysiol. 2018;129(12):2534–40. 30384023 10.1016/j.clinph.2018.10.001

[75] Kouamou Ntonfo G, Ferrari G, Lofino F, Raheli R, Pisani F, Extraction of video features for real-time detection of neonatal seizures. IEEE International Symposium on a World of Wireless. Mobile Multimedia Netw. 2011;2011:1–6.

[76] Ntonfo G, Lofino F, Ferrari G, Raheli R, Pisani F. Video processing-based detection of neonatal seizures by trajectory features clustering. IEEE Int Conf Commun. 2012:3456–60.

[77] Pisani F, Spagnoli C, Pavlidis E, Facini C, Kouamou Ntonfo GM, Ferrari G, et al. Real-time automated detection of clonic seizures in newborns. Clin Neurophysiol. 2014;125(8):1533–40. 24602566 10.1016/j.clinph.2013.12.119

[78] Cattani L, Alinovi D, Ferrari G, Raheli R, Pavlidis E, Spagnoli C, et al. Monitoring infants by automatic video processing: a unified approach to motion analysis. Comput Biol Med. 2017;80:158–65. 27940321 10.1016/j.compbiomed.2016.11.010

[79] Karayiannis NB, Srinivasan S, Bhattacharya R, Wise M, Frost JD Jr, Mizrahi EM. Extraction of motion strength and motor activity signals from video recordings of neonatal seizures. IEEE Trans Med Imaging. 2011;20(9):965–80. 10.1109/42.952733 11585212

[80] Karayiannis NB, Tao G. Improving the extraction of temporal motion strength signals from video recording of neonatal seizures. IEEE Conference on Advanced Video and Signal Based Surveillance. 2003:87–92.

[81] Karayiannis NB, Tao G. An improved procedure for the extraction of temporal motion strength signals from video recordings of neonatal seizures. Image Vision Comput. 2006;24(1):27–40.

[82] Karayiannis NB, Tao G, Xiong Y, Sami A, Varughese B, Frost JD Jr, et al. Computerized motion analysis of videotaped neonatal seizures of epileptic origin. Epilepsia. 2005;46(6):901–17. 15946330 10.1111/j.1528-1167.2005.56504.x

[83] Lawrence R, Mathur A, Nguyen TheTich S, Zempel J, Inder T. A pilot study of continuous limited-channel aEEG in term infants with encephalopathy. J Pediatr. 2009;154(6):835–41. 19230897 10.1016/j.jpeds.2009.01.002

[84] Gotman J, Flanagan D, Rosenblatt B, Bye A, Mizrahi EM. Evaluation of an automatic seizure detection method for the newborn EEG. Electroencephalogr Clin Neurophysiol. 1997;103(3):363–9. 9305283 10.1016/s0013-4694(97)00005-2

[85] Navakatikyan MA, Colditz PB, Burke CJ, Inder TE, Richmond J, Williams CE. Seizure detection algorithm for neonates based on wave-sequence analysis. Clin Neurophysiol. 2006;117(6):1190–203. 16621690 10.1016/j.clinph.2006.02.016

[86] Gomez-Quintana S, O’Shea A, Factor A, Popovici E, Temko A. A method for AI assisted human interpretation of neonatal EEG. Sci Rep. 2022;12(1):10932. 35768501 10.1038/s41598-022-14894-4 PMC9243143

[87] Dilena R, De Liso P, Di Capua M, Consonni D, Capovilla G, Pisani F, et al. Influence of etiology on treatment choices for neonatal seizures: A survey among pediatric neurologists. Brain Dev. 2019;41(7):595–9. 30954359 10.1016/j.braindev.2019.03.012

[88] Painter MJ, Scher MS, Stein AD, Armatti S, Wang Z, Gardiner JC, et al. Phenobarbital compared with phenytoin for the treatment of neonatal seizures. N Engl J Med. 1999;341(7):485–9. 10441604 10.1056/NEJM199908123410704

[89] Sharpe C, Reiner GE, Davis SL, Nespeca M, Gold JJ, Rasmussen M, et al. Levetiracetam versus phenobarbital for neonatal seizures: a randomized controlled trial. Pediatrics. 2020;145(6):e20193182. 32385134 10.1542/peds.2019-3182 PMC7263056

[90] Ramantani G, Pisani F. Neonatal seizures-diagnostic options and treatment recommendations. Z Epileptol. 2022;35(4):310–6.

[91] Bittigau P, Sifringer M, Genz K, Reith E, Pospischil D, Govindarajalu S, et al. Antiepileptic drugs and apoptotic neurodegeneration in the developing brain. Proc Natl Acad Sci USA. 2002;99(23):15089–94. 12417760 10.1073/pnas.222550499 PMC137548

[92] Forcelli PA, Janssen MJ, Vicini S, Gale K. Neonatal exposure to antiepileptic drugs disrupts striatal synaptic development. Ann Neurol. 2012;72(3):363–72. 22581672 10.1002/ana.23600 PMC3421036

[93] Silverstein FS, Ferriero DM. Off-label use of antiepileptic drugs for the treatment of neonatal seizures. Pediatr Neurol. 2008;39(2):77–9. 18639748 10.1016/j.pediatrneurol.2008.04.008

[94] Mruk AL, Garlitz KL, Leung NR. Levetiracetam in neonatal seizures: a review. J Pediatr Pharmacol Ther. 2015;20(2):76–89. 25964725 10.5863/1551-6776-20.2.76 PMC4418685

[95] Ramantani G, Ikonomidou C, Walter B, Rating D, Dinger J. Levetiracetam: safety and efficacy in neonatal seizures. Eur J Paediatr Neurol. 2011;15(1):1–7. 21094062 10.1016/j.ejpn.2010.10.003

[96] Falsaperla R, Vitaliti G, Mauceri L, Romano C, Pavone P, Motamed-Gorji N, et al. Levetiracetam in neonatal seizures as first-line treatment: a prospective study. J Pediatr Neurosci. 2017;12(1):24–8. 28553374 10.4103/jpn.JPN_172_16 PMC5437782

[97] Hooper RG, Ramaswamy VV, Wahid RM, Satodia P, Bhulani A. Levetiracetam as the first-line treatment for neonatal seizures: a systematic review and meta-analysis. Dev Med Child Neurol. 2021;63(11):1283–93. 34124790 10.1111/dmcn.14943

[98] Rondagh M, De Vries LS, Peeters-Scholte CMPCD, Tromp SC, Steggerda SJ. Efficacy of Levetiracetam as Add-On Therapy in the Treatment of Seizures in Neonates. Neonatology. 2023:1–11. 10.1159/000535499 PMC10994567 38113859

[99] Boylan GB, Rennie JM, Chorley G, Pressler RM, Fox GF, Farrer K, et al. Second-line anticonvulsant treatment of neonatal seizures: a video-EEG monitoring study. Neurology. 2004;62(3):486–8. 14872039 10.1212/01.wnl.0000106944.59990.e6

[100] Pressler RM, Abend NS, Auvin S, Boylan G, Brigo F, Cilio MR, et al. Treatment of seizures in the neonate: Guidelines and consensus-based recommendations-Special report from the ILAE Task Force on Neonatal Seizures. Epilepsia. 2023;64(10):2550–70. 37655702 10.1111/epi.17745

[101] Abiramalatha T, Thanigainathan S, Ramaswamy VV, Pressler R, Brigo F, Hartmann H. Anti-seizure medications for neonates with seizures. Cochrane Database Syst Rev. 2023;10(10):CD014967. 37873971 10.1002/14651858.CD014967.pub2 PMC10594593

[102] Glass HC, Nash KB, Bonifacio SL, Barkovich AJ, Ferriero DM, Sullivan JE, et al. Seizures and magnetic resonance imaging-detected brain injury in newborns cooled for hypoxic-ischemic encephalopathy. J Pediatr. 2011;159(5):731–735.e1. 21839470 10.1016/j.jpeds.2011.07.015 PMC3193544

[103] Pisani F, Cerminara C, Fusco C, Sisti L. Neonatal status epilepticus vs recurrent neonatal seizures: clinical findings and outcome. Neurology. 2007;69(23):2177–85. 18056582 10.1212/01.wnl.0000295674.34193.9e

[104] Ramantani G. Neonatal epilepsy and underlying aetiology: to what extent do seizures and EEG abnormalities influence outcome? Epileptic Disord. 2013;15(4):365–75. 24342861 10.1684/epd.2013.0619

[105] Kharoshankaya L, Stevenson NJ, Livingstone V, Murray DM, Murphy BP, Ahearne CE, et al. Seizure burden and neurodevelopmental outcome in neonates with hypoxic-ischemic encephalopathy. Dev Med Child Neurol. 2016;58(12):1242–8. 27595841 10.1111/dmcn.13215 PMC5214689

[106] Miller SP, Weiss J, Barnwell A, Ferriero DM, Latal-Hajnal B, Ferrer-Rogers A, et al. Seizure-associated brain injury in term newborns with perinatal asphyxia. Neurology. 2002;58(4):542–8. 11865130 10.1212/wnl.58.4.542

[107] Jensen FE. Acute and chronic effects of seizures in the developing brain: experimental models. Epilepsia. 1999;40:S51–8. 10421561 10.1111/j.1528-1157.1999.tb00879.x

[108] Trowbridge SK, Condie LO, Landers JR, Bergin AM, Grant PE, Krishnamoorthy K, et al. Effect of neonatal seizure burden and etiology on the long-term outcome: data from a randomized, controlled trial. Ann Child Neurol Soc. 2023;1(1):53–65. 37636014 10.1002/cns3.8 PMC10449023

[109] Stevenson NJ, Vanhatalo S. Designing a trial for neonatal seizure treatment. Semin Fetal Neonatal Med. 2018;23(3):213–7. 29467103 10.1016/j.siny.2018.02.005

[110] Bättig L, Dünner C, Cserpan D, Rüegger A, Hagmann C, Schmitt B, et al. Levetiracetam versus phenobarbital for neonatal seizures: a retrospective cohort study. Pediatr Neurol. 2023;138:62–70. 36401982 10.1016/j.pediatrneurol.2022.10.004

[111] Spagnoli C, Seri S, Pavlidis E, Mazzotta S, Pelosi A, Pisani F. Phenobarbital for neonatal seizures: response rate and predictors of refractoriness. Neuropediatrics. 2016;47(5):318–26. 27458678 10.1055/s-0036-1586214

[112] Dwivedi D, Lin N, Venkatesan C, Kline-Fath B, Holland K, Schapiro M. Clinical, neuroimaging, and electrographicpredictors of phenobarbital failure in newborns with hypoxic ischemic encephalopathy and seizures. J Child Neurol. 2019;34(8):458–63. 30966848 10.1177/0883073819838171

[113] Pisani F, Sisti L, Seri S. A scoring system for early prognostic assessment after neonatal seizures. Pediatrics. 2009;124(4):e580–7. 19752080 10.1542/peds.2008-2087

[114] Mellits ED, Holden KR, Freeman JM. Neonatal seizures. II. A multivariate analysis of factors associated with outcome. Pediatrics. 1982;70(2):177–85. 7099783

[115] Glass HC, Grinspan ZM, Shellhaas RA. Outcomes after acute symptomatic seizures in neonates. Semin Fetal Neonatal Med. 2018;23(3):218–22. 29454756 10.1016/j.siny.2018.02.001

[116] Glass HC, Shellhaas RA, Tsuchida TN, Chang T, Wusthoff CJ, Chu CJ, et al. Seizures in preterm neonates: a multicenter observational cohort study. Pediatr Neurol. 2017;72:19–24. 28558955 10.1016/j.pediatrneurol.2017.04.016 PMC5863228

[117] Davis AS, Hintz SR, Van Meurs KP, Li L, Das A, Stoll BJ, et al. Seizures in extremely low birth weight infants are associated with adverse outcome. J Pediatr. 2010;157(720–5):e1-2. 10.1016/j.jpeds.2010.04.065 PMC2939969 20542294

[118] Pisani F, Copioli C, Turco EC, Sisti L, Cossu G, Seri S. Mortality risk after neonatal seizures in very preterm newborns. J Child Neurol. 2012;27:1264–9. 22378670 10.1177/0883073811435244

[119] Pisani F, Prezioso G, Spagnoli C. Neonatal seizures in preterm infants: A systematic review of mortality risk and neurological outcomes from studies in the 2000’s. Seizure. 2020;75:7–17. 31864147 10.1016/j.seizure.2019.12.005

[120] Pisani F, Spagnoli C. Outcome in preterm infants with seizures. Handb Clin Neurol (3rd series). 2019;162:401–14. 10.1016/B978-0-444-64029-1.00019-9 31324322

[121] Schüssler SC, Schmidt M, Deiters L, Candova A, Fahlbusch FB, Trollmann R. Long-term outcomes of very-low-birth-weight and low-birth-weight preterm newborns with neonatal seizures: a single-center perspective. Eur J Paediatr Neurol. 2022;36:137–42. 34973622 10.1016/j.ejpn.2021.12.013

[122] Ronen GM, Buckley D, Penney S, Streiner DL. Long-term prognosis in children with neonatal seizures: a population-based study. Neurology. 2007;69(19):1816–22. 17984448 10.1212/01.wnl.0000279335.85797.2c

[123] Scher MS, Aso K, Beggarly ME, Hamid MY, Steppe DA, Painter MJ. Electrographic seizures in preterm and full-term neonates: clinical correlates, associated brain lesions, and risk for neurologic sequelae. Pediatrics. 1993;91(1):128–34. 8416475

[124] Pisani F, Piccolo B, Cantalupo G, Copioli C, Fusco C, Pelosi A, et al. Neonatal seizures and postneonatal epilepsy: a 7-y follow-up study. Pediatr Res. 2012;72(2):186–93. 22580721 10.1038/pr.2012.66

[125] Pisani F, Facini C, Pavlidis E, Spagnoli C, Boylan G. Epilepsy after neonatal seizures: literature review. Eur J Paediatr Neurol. 2015;19(1):6–14. 25455712 10.1016/j.ejpn.2014.10.001

[126] Pisani F, Pavlidis E, Facini C, La Morgia C, Fusco C, Cantalupo G. A 15-year epileptogenic period after perinatal brain injury. Funct Neurol. 2017;32(1):49–53. 28380324 10.11138/FNeur/2017.32.1.049 PMC5505530

[127] Suppiej A, Mastrangelo M, Mastella L, Accorsi P, Grazian L, Casara G, et al. Pediatric epilepsy following neonatal seizures symptomatic of stroke. Brain Dev. 2016;38(1):27–31. 26058328 10.1016/j.braindev.2015.05.010

[128] Pisani F, Fusco C, Nagarajan L, Spagnoli C. Acute symptomatic neonatal seizures, brain injury, and long-term outcome: the role of neuroprotective strategies. Expert Rev Neurother. 2021;21(2):189–203. 33176104 10.1080/14737175.2021.1848547

[129] Tagin MA, Woolcott CG, Vincer MJ, Whyte RK, Stinson DA. Hypothermia for neonatal hypoxic ischemic encephalopathy: an updated systematic review and meta-analysis. Arch Pediatr Adolesc Med. 2012;166(6):558. 22312166 10.1001/archpediatrics.2011.1772

[130] Martinello K, Hart AR, Yap S, Mitra S, Robertson NJ. Management and investigation of neonatal encephalopathy: 2017 update. Arch Dis Child Fetal Neonatal Ed. 2017;102(4):F346. 28389438 10.1136/archdischild-2015-309639 PMC5537522

[131] Pappas A, Shankaran S, McDonald SA, Vohr BR, Hintz SR, Ehrenkranz RA, et al. Cognitive outcomes after neonatal encephalopathy. Pediatrics. 2015;135(3):e624–34. 25713280 10.1542/peds.2014-1566 PMC4338321

[132] Amare HT, Amare AT. Etiology, clinical features, and short-term outcome of seizures in newborns admitted to the university of gondar hospital Ethiopia. Pediatric Health Med Ther. 2019;10:107–13. 31695557 10.2147/PHMT.S228241 PMC6804667

[133] Mwaniki M, Mathenge A, Gwer S, Mturi N, Bauni E, Newton CR, et al. Neonatal seizures in a rural Kenyan District Hospital: aetiology, incidence and outcome of hospitalization. BMC Med. 2010;8:16. 20236524 10.1186/1741-7015-8-16 PMC2846860

[134] Garg A, Suthar R, Sundaram V, Kumar P, Angurana SK. Clinical profile, aetiology, short-term outcome and predictors of poor outcome of neonatal seizures among out-born neonates admitted to a neonatal unit in Paediatric emergency of a tertiary care hospital in North India: A prospective observational study. Trop Doct. 2021;51(3):365–71. 34018889 10.1177/00494755211016226

[135] Weldegerima K, Gebremariam DS, Haftu H, Berhe G, Hadgu A, Mohammedamin MM. Neonatal seizure pattern, outcome, and its predictors among neonates admitted to NICU of Ayder comprehensive specialized hospital, Mekelle, Tigray Ethiopia. Int J Gen Med. 2023;16:4343–55. 37781273 10.2147/IJGM.S414420 PMC10540696

[136] Sabzehei MK, Basiri B, Bazmamoun H. The Etiology, Clinical Type, and Short Outcome of Seizures in NewbornsHospitalized in Besat Hospital/Hamadan/ Iran. Iran J Child Neurol. 2014;8(2):24–8. 24949047 PMC4058061

[137] Al-Momen H, Muhammed MK, Alshaheen AA. Neonatal seizures in Iraq: cause and outcome. Tohoku J Exp Med. 2018;246(4):245–9. 30555128 10.1620/tjem.246.245

[138] Lai YH, Ho CS, Chiu NC, Tseng CF, Huang YL. Prognostic factors of developmental outcome in neonatal seizures in term infants. Pediatr Neonatol. 2013;54(3):166–72. 23597533 10.1016/j.pedneo.2013.01.001

[139] Uria-Avellanal C, Marlow N, Rennie JM. Outcome following neonatal seizures. Semin Fetal Neonatal Med. 2013;18(4):224–32. 23466296 10.1016/j.siny.2013.01.002

[140] Pisani F, Barilli AM, Sisti L, Bevilacqua G, Seri S. Preterm infants with video-EEG confirmed seizures: Outcome at 30 months of age. Brain Dev. 2008;30(1):20–30. 17964748 10.1016/j.braindev.2007.05.003

[141] Pisani F, Copioli C, Di Gioia C, Turco E, Sisti L. Neonatal seizures: Relation of ictal videoelectroencephalography findings with neurodevelopmental outcome. J Child Neurol. 2008;23:394–8. 18192647 10.1177/0883073807309253

[142] Pisani F, Facini C, Pelosi A, Mazzotta S, Spagnoli C, Pavlidis E. Neonatal seizures in preterm newborns: a predictive model for outcome. Eur J Paediatr Neurol. 2016;20(2):243–51. 26777334 10.1016/j.ejpn.2015.12.007

[143] Twomey E, Twomey A, Ryan S, Murphy J, Donoghue VB. MR imaging of term infants with hypoxic-ischaemic encephalopathy as a predictor of neurodevelopmental outcome and late MRI appearances. Pediatr Radiol. 2010;40(9):1526–35. 20512322 10.1007/s00247-010-1692-9

[144] Martinez-Biarge M, Madero R, González A, Quero J, García-Alix A. Perinatal morbidity and risk of hypoxic-ischemic encephalopathy associated with intrapartum sentinel events. Am J Obstet Gynecol. 2012;206(2):148.e1-148.e7. 22079054 10.1016/j.ajog.2011.09.031

[145] Harteman JC, Groenendaal F, Toet MC, Benders MJ, Van Haastert IC, Nievelstein RA, et al. Diffusion-weighted imaging changes in cerebral watershed distribution following neonatal encephalopathy are not invariably associated with an adverse outcome. Dev Med Child Neurol. 2013;55(7):642–53. 23550687 10.1111/dmcn.12122

[146] Miller SP, Ramaswamy V, Michelson D, Barkovich AJ, Holshouser B, Wycliffe N, et al. Patterns of brain injury in term neonatal encephalopathy. J Pediatr. 2005;146(4):453–60. 15812446 10.1016/j.jpeds.2004.12.026

[147] Sato Y, Hayakawa M, Iwata O, Okumura A, Kato T, Hayakawa F, et al. Delayed neurological signs following isolated parasagittal injury in asphyxia at term. Eur J Paediatr Neurol. 2008;12(5):359–65. 18054507 10.1016/j.ejpn.2007.10.003

[148] van Rooij LG, de Vries LS, Handryastuti S, Hawani D, Groenendaal F, van Huffelen AC, et al. Neurodevelopmental outcome in term infants with status epilepticus detected with amplitude-integrated electroencephalography. Pediatrics. 2007;120(2):e354–63. 17671044 10.1542/peds.2006-3007

[149] Altınbezer P, Çolak R, Çalkavur Ş, Yılmaz Ü. Epilepsy frequency and risk factors three years after neonatal seizures. Pediatr Neurol. 2023;149:120–6. 37866139 10.1016/j.pediatrneurol.2023.09.015

[150] Maartens IA, Wassenberg T, Buijs J, Bok L, de Kleine MJ, Katgert T, et al. Neurodevelopmental outcome in full-term newborns with refractory neonatal seizures. Acta Paediatr. 2012;101(4):e173–8. 22085256 10.1111/j.1651-2227.2011.02528.x

[151] Basti C, Maranella E, Cimini N, Catalucci A, Ciccarelli S, Del Torto M. Seizure burden and neurodevelopmental outcome in newborns with hypoxic-ischemic encephalopathy treated with therapeutic hypothermia: A single center observational study. Seizure. 2020;83:154–9. 33160202 10.1016/j.seizure.2020.10.021

[152] van der Heide MJ, Roze E, van der Veere CN, Ter Horst HJ, Brouwer OF, Bos AF. Long-term neurological outcome of term-born children treated with two or more anti-epileptic drugs during the neonatal period. Early Hum Dev. 2012;88(1):33–8. 21835564 10.1016/j.earlhumdev.2011.06.012

